# Cabozantinib-associated therapy-refractory lower-leg ulcers in metastatic renal cell carcinoma

**DOI:** 10.1016/j.jdcr.2026.07.048

**Published:** 2026-08-01

**Authors:** Zhide Meng, Tobias M. Franz, Benedikt Hindelang, Susanne A. Steimle-Grauer, Tilo Biedermann

**Affiliations:** Department of Dermatology and Allergy Biederstein, School of Medicine and Health, Technical University of Munich, Munich, Germany

**Keywords:** adverse effects, cabozantinib, chronic ulceration, dermatologic toxicity, multi-kinase inhibitor, pyoderma gangrenosum, renal cell carcinoma, VEGF, VEGFR inhibition, wound management

## Introduction

**Fig 1:**
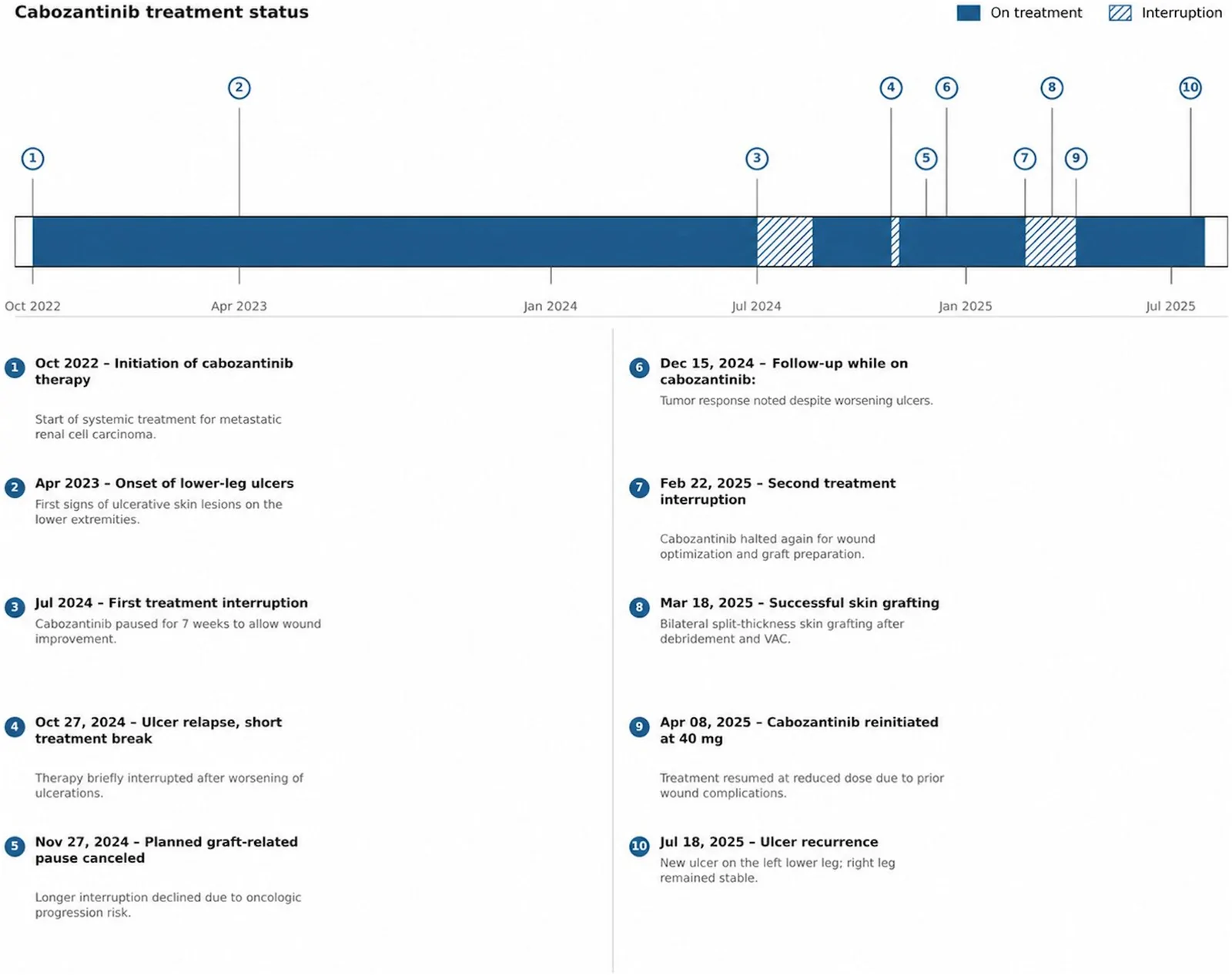
A Chronological summary of oncologic and wound management milestones: Cabozantinib therapy and chronic leg ulcer management.

Receptor tyrosine kinases (RTKs) regulate proliferation, differentiation, survival, and metabolism. Dysregulation of these pathways, particularly vascular endothelial growth factor (VEGF) signaling, contributes to angiogenesis and progression in renal cell carcinoma (RCC).^1^^,^^2^ Cabozantinib, a potent multi-kinase inhibitor targeting VEGFR, MET, and AXL, is effective in RCC and hepatocellular carcinoma.^3^, ^4^, ^5^ While VEGF inhibition is crucial for cabozantinib’s antitumor effect, it also leads to significant adverse effects, particularly affecting the skin and mucosa, leading to hand-foot skin reaction (HFSR), hypopigmentation, and gastrointestinal ulcerations. Rarely, scrotal ulcerations have also been reported.^6^^,^^7^ We report a patient with chronic, therapy-refractory lower leg ulcerations associated with venous insufficiency and treated with cabozantinib and discuss the underlying pathophysiology as well as clinical implications for management (Fig 1).

## Case report

The patient was diagnosed with metastatic renal cell carcinoma in July 2022 following CT-guided biopsy of a pelvic bone lesion, which revealed osseous, lymphatic, adrenal, hilar, and mediastinal metastases. Histopathological analysis suggested clear-cell RCC, though an exact subclassification was not possible. Based on International Metastatic Renal Cell Carcinoma Database Consortium (IMDC) criteria, the patient was classified as Stage IV with intermediate risk.^8^ First-line therapy with ipilimumab and nivolumab began in August 2022 but was halted after 4 cycles due to a pronounced maculopapular immune-related cutaneous adverse event (irCAE) (CTCAE grade 3-4). Despite subsequent stereotactic radiotherapy (35 Gy), tumor progression was observed in October 2022. Consequently, cabozantinib (60 mg/day) was initiated and led to partial response with regression of the primary tumor and metastases.

**Fig 2:**
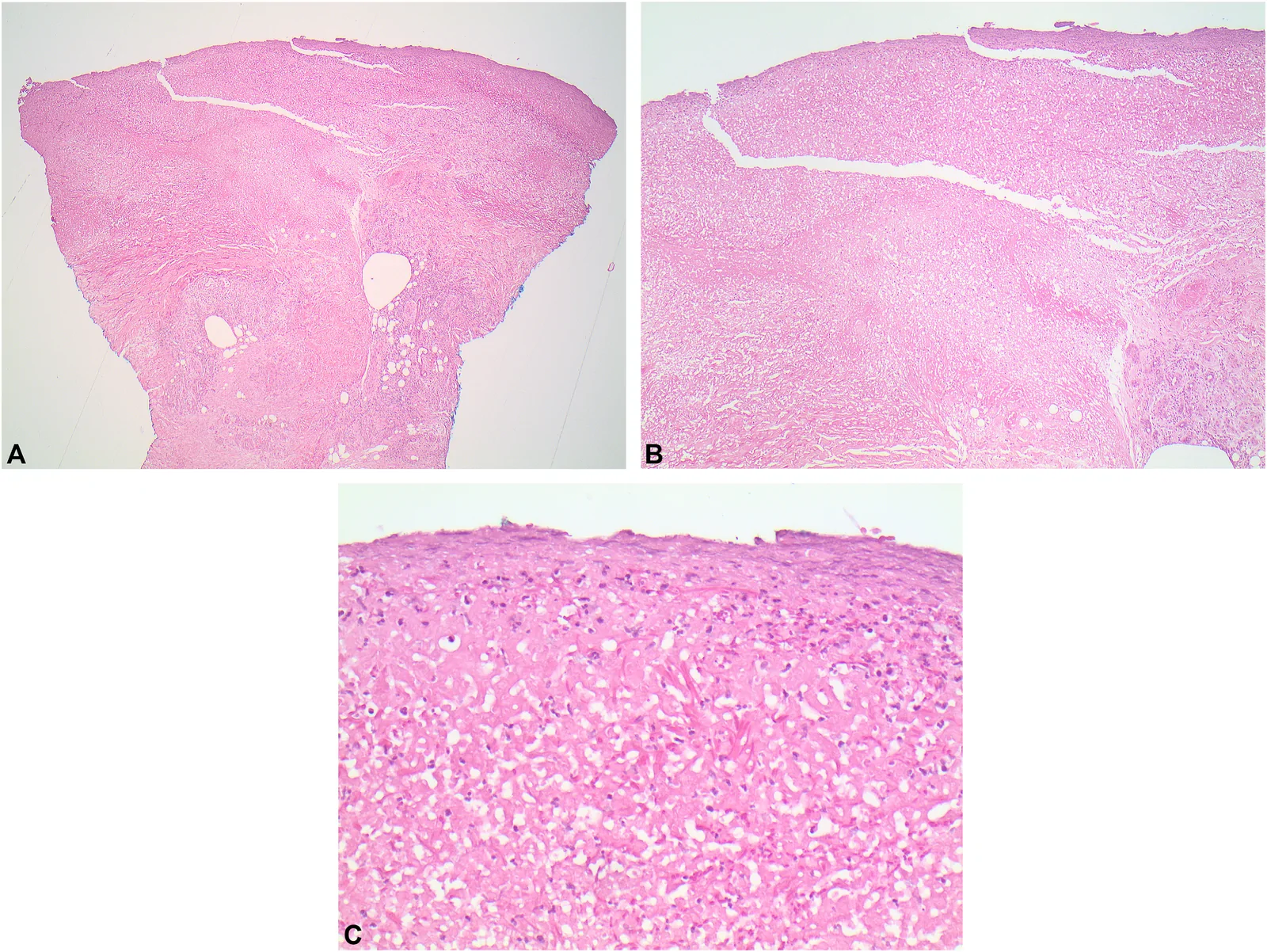
Histopathology of ulcer biopsy. **A-C,** Histopathology of ulcer biopsy showing necrotic areas of epidermis, dermis, and subcutis with a neutrophil-rich inflammation pattern (hematoxylin–eosin staining, ×1, ×4, and ×20).

**Fig 3:**
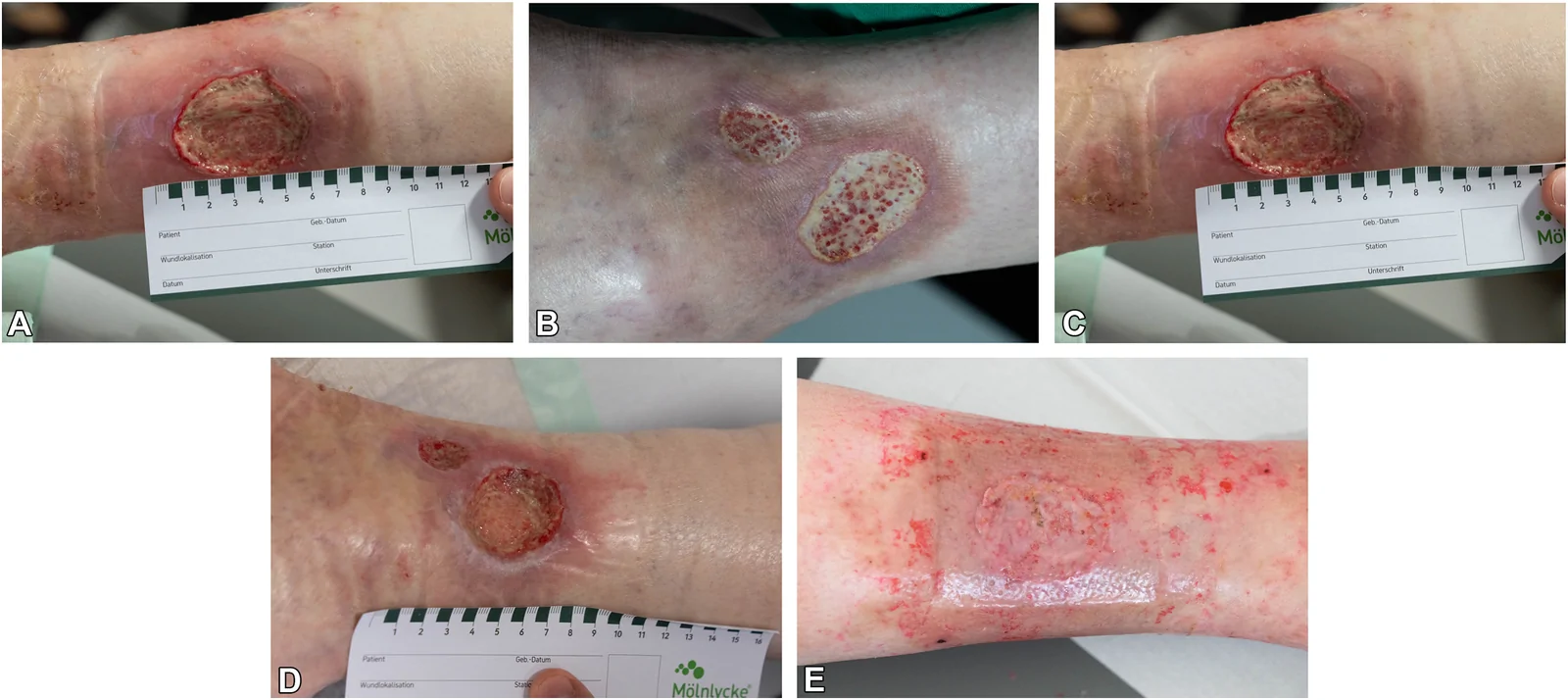
Clinical course of bilateral lower-leg ulcers. **A,** Right lower leg ulcer in August 2024 showing partial granulation after a 7-week interruption of cabozantinib and intensive wound care. **B,** Left lower leg ulcer in August 2024 showing partial granulation after a 7-week interruption of cabozantinib and intensive wound care. **C,** Right lower leg ulcer after treatment resumption, showing marked deterioration with fibrin coverage **(approximately 5 × 5 cm)**. **D,** Left lower leg ulcer after treatment resumption, showing marked deterioration with fibrin coverage **(approximately 4 × 4 cm; depth 2-10 mm)**. **E,** Postoperative appearance of the left lower leg 20 days after split-thickness skin grafting, showing good graft adherence and no signs of infection.

The patient had asymptomatic chronic venous insufficiency without compression therapy, as well as arterial hypertension, hypothyroidism, and nephrolithiasis. He had no history of cutaneous ulcers, peripheral arterial occlusive disease, or diabetes mellitus. Six months after starting cabozantinib, the patient self-reported a small cutaneous ulcer on his right leg, which developed spontaneously and was managed conservatively. In May 2023, the patient experienced side effects like diarrhea and polyneuropathy, while the oncologic course remained stable. By July 2024, the painful ulceration deteriorated, raising suspicion of cabozantinib toxicity, so cabozantinib treatment was paused. Routine wound swabs showed only normal skin flora (coagulase-negative staphylococci, viridans streptococci, and non-pathogenic corynebacteria). Biopsy showed ulceration with an underlying neutrophilic inflammation pattern, suggesting pyoderma gangrenosum (PG). Although anti-neutrophilic therapies such as dapsone were considered, they were not initiated because the clinicopathologic findings did not support a definitive diagnosis of PG. Malignancy was excluded (Fig 2). After 7 weeks of compression and intensive wound care without cabozantinib, including dressing changes every 1–2 days with soft silicone-coated polyamide wound contact layer and a polyhexanide-containing hydrogel based on Ringer solution, the ulcers showed granulation and partial re-epithelization (Fig 3). However, 2 months after reinitiation, ulcers worsened, leading to two deep lesions on the left lower leg and a third ulcer on the right lower leg. Cabozantinib was paused (October 27-November 6, 2024) and vacuum-assisted closure therapy led to reduction of wound size and regranulation. A planned longer interruption until early December for split-thickness grafting was declined because treatment breaks beyond 4 weeks were felt to carry an unacceptable risk of progression. Cabozantinib was continued, resulting in marked regression of adrenal and hilar lung metastases by December 2024, but also in renewed worsening of the ulcerations.

In light of the now stabilized oncologic situation, a longer interruption of cabozantinib therapy was undertaken, from February 22 to April 8, 2025, to optimize conditions for skin grafting. At admission, the ulcers were well-defined, fibrin-covered, and measured approximately 5 × 5 cm on the right leg and 4 × 4 cm on the left, with depths ranging from 2 to 10 mm (Fig 3). Between February 25 and March 14, the patient underwent 6 cycles of surgical debridement and VAC therapy, with each VAC dressing changed every 4-5 days, followed by successful bilateral split-thickness skin grafting on March 18. Postoperative healing was uncomplicated, with good graft adherence and no infection. Wound care included an additional 2 days of VAC support, followed by regular dressing changes and compression therapy.

During hospitalization, asymptomatic gross hematuria prompted cystoscopy, which revealed two calcified exophytic bladder tumors. Immunohistochemistry (PAX8+, pan-CK+) was consistent with a metastatic manifestation of the known renal cell carcinoma.

Cabozantinib was reinitiated at a reduced dose of 40 mg on April 8, 2025. Restaging in July showed no new metastases and only mild primary tumor progression. After reinitiation, a new ulcer developed on the left lower leg, while the right leg remained clinically stable.

## Discussion

This case highlights both the importance of recognizing chronic lower-leg ulcerations as a potential adverse effect of cabozantinib and the clinical challenge of managing these ulcers in a patient with preexisting venous insufficiency. The temporal course strongly suggests that cabozantinib played a substantial role in ulcer development and persistence: the ulcers consistently improved during treatment interruption, worsened after re-exposure, and showed partial recurrence even after successful surgical management and dose reduction.

Reports of cabozantinib-associated lower-leg ulcers in patients with venous insufficiency and a similarly chronic, therapy-refractory course are scarce. Several mechanisms may contribute to chronic cutaneous ulcerations. First, VEGFR inhibition leads to endothelial dysfunction and microvascular impairment, thereby compromising wound healing.^9^^,^^10^ Second, cabozantinib's immunomodulatory effects might lead to a prolonged inflammatory response and poor tissue regeneration,^10^ contributing to ulcer development and persistence. Third, the patient's history of chronic venous insufficiency may have predisposed him to impaired wound healing, further exacerbated by cabozantinib.

Although the histology suggested PG, it was not confirmed clinically. However, the clinical presentation bears similarities to PG-like ulcerations under cabozantinib therapy, as reported by Matsudate et al (2022), highlighting a possible link between VEGFR inhibition and neutrophilic dermatoses.^11^

In conclusion, this case highlights a severe and previously underrecognized adverse event associated with cabozantinib therapy. Given the increasing use of VEGFR-targeted TKIs such as cabozantinib, clinicians should remain vigilant for rare but clinically significant dermatologic toxicities, particularly in patients with risk factors such as chronic venous insufficiency, peripheral arterial disease, and diabetes mellitus. Because balancing oncologic control with wound management remains challenging, especially when treatment interruptions may increase the risk of tumor progression, early dermatologic involvement, individualized risk-benefit assessment, and multidisciplinary treatment strategies are essential to achieving optimal oncologic and dermatologic outcomes.

## Declaration of generative AI and AI-assisted technologies in the writing process

During the preparation of this work, the authors used ChatGPT (GPT-5.5 Thinking, OpenAI, San Francisco, CA, USA) solely for language editing, grammar correction, and improvement of readability in selected passages. It was not used to generate scientific content, analyze data, interpret the results, or formulate conclusions. The authors reviewed and edited the output as needed and take full responsibility for the content of the published article.

## Conflicts of interest

None disclosed.
